# Predicting post-contrast information from contrast agent free cardiac MRI using machine learning: Challenges and methods

**DOI:** 10.3389/fcvm.2022.894503

**Published:** 2022-07-27

**Authors:** Musa Abdulkareem, Asmaa A. Kenawy, Elisa Rauseo, Aaron M. Lee, Alireza Sojoudi, Alborz Amir-Khalili, Karim Lekadir, Alistair A. Young, Michael R. Barnes, Philipp Barckow, Mohammed Y. Khanji, Nay Aung, Steffen E. Petersen

**Affiliations:** ^1^Barts Heart Centre, Barts Health National Health Service (NHS) Trust, London, United Kingdom; ^2^National Institute for Health Research (NIHR) Barts Biomedical Research Centre, William Harvey Research Institute, Queen Mary University of London, London, United Kingdom; ^3^Health Data Research UK, London, United Kingdom; ^4^Circle Cardiovascular Imaging Inc., Calgary, AB, Canada; ^5^Artificial Intelligence in Medicine Lab (BCN-AIM), Faculty of Mathematics and Computer Science, University of Barcelona, Barcelona, Spain; ^6^Department of Biomedical Engineering, King’s College London, London, United Kingdom; ^7^Centre for Translational Bioinformatics, William Harvey Research Institute, Faculty of Medicine and Dentistry, Queen Mary University of London, London, United Kingdom; ^8^Newham University Hospital, Barts Health National Health Service (NHS) Trust, London, United Kingdom; ^9^The Alan Turing Institute, London, United Kingdom

**Keywords:** CMR, contrast, contrast-free, deep learning, machine learning, support vector machines, decision tree

## Abstract

**Objectives:**

Currently, administering contrast agents is necessary for accurately visualizing and quantifying presence, location, and extent of myocardial infarction (MI) with cardiac magnetic resonance (CMR). In this study, our objective is to investigate and analyze pre- and post-contrast CMR images with the goal of predicting post-contrast information using pre-contrast information only. We propose methods and identify challenges.

**Methods:**

The study population consists of 272 retrospectively selected CMR studies with diagnoses of MI (*n* = 108) and healthy controls (*n* = 164). We describe a pipeline for pre-processing this dataset for analysis. After data feature engineering, 722 cine short-axis (SAX) images and segmentation mask pairs were used for experimentation. This constitutes 506, 108, and 108 pairs for the training, validation, and testing sets, respectively. We use deep learning (DL) segmentation (UNet) and classification (ResNet50) models to discover the extent and location of the scar and classify between the ischemic cases and healthy cases (i.e., cases with no regional myocardial scar) from the pre-contrast cine SAX image frames, respectively. We then capture complex data patterns that represent subtle signal and functional changes in the cine SAX images due to MI using optical flow, rate of change of myocardial area, and radiomics data. We apply this dataset to explore two supervised learning methods, namely, the support vector machines (SVM) and the decision tree (DT) methods, to develop predictive models for classifying pre-contrast cine SAX images as being a case of MI or healthy.

**Results:**

Overall, for the UNet segmentation model, the performance based on the mean Dice score for the test set (*n* = 108) is 0.75 (±0.20) for the endocardium, 0.51 (±0.21) for the epicardium and 0.20 (±0.17) for the scar. For the classification task, the accuracy, F1 and precision scores of 0.68, 0.69, and 0.64, respectively, were achieved with the SVM model, and of 0.62, 0.63, and 0.72, respectively, with the DT model.

**Conclusion:**

We have presented some promising approaches involving DL, SVM, and DT methods in an attempt to accurately predict contrast information from non-contrast images. While our initial results are modest for this challenging task, this area of research still poses several open problems.

## Introduction

### Background and objectives

Cardiovascular diseases (CVDs) are a major cause of death in the world in 2022, causing approximately 18.6 million deaths (31% of all deaths) annually according to the World Heart Federation (1). Ischemic heart disease (IHD) was responsible for almost half of all cardiac deaths in 2019 (2). A common and important consequence of IHD is myocardial infarction (MI) defined pathologically as myocardial cell death due to prolonged ischemia which can lead to the loss of contraction of that damaged portion of the heart muscle.

**FIGURE 1:**
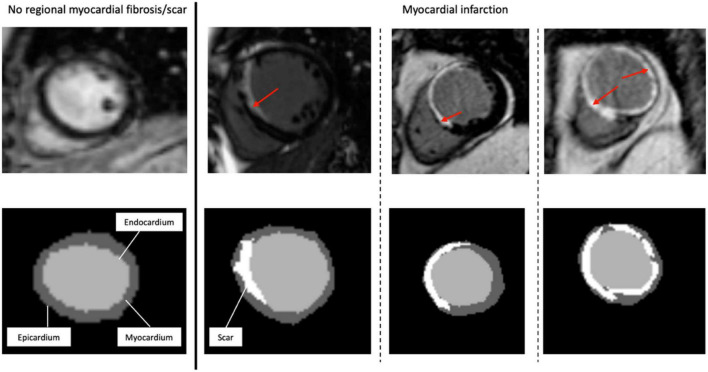
Representative short-axis late gadolinium enhancement-cardiac magnetic resonance images (LGE-CMR) of various possible locations and extents of myocardial scar. The top images are the original LGE images, with the red arrows indicating the distribution of LGE (scar) in the LV wall segments. At the bottom are the segmentation masks (expert manual contouring) with the scarred regions in white.

Cardiac magnetic resonance (CMR) is an imaging modality that has proven to be very effective in diagnosing MI through visualization of the regional myocardial scar allowing the determination of the presence, location, and extent. Currently, administering contrast agent, using gadolinium-based chelates (gadolinium), is necessary for diagnosing MI with CMR. This technique relies on the relative gadolinium accumulation in areas of necrosis and fibrosis following myocardial damage. The presence and pattern of the gadolinium contrast can vary, with subendocardial or transmural late gadolinium enhancement (LGE) images usually indicating fibrosis caused by previous coronary ischemic events or MI. Eliminating the need of contrast administration could in several ways benefit many patients, such as, patients who cannot be safely given a contrast agent due to allergies or severe kidney disease, and could improve safety and patient experience (avoiding need for intravenous cannulation) and costs of cardiovascular healthcare. Moreover, a typical contrast CMR scan takes approximately 35–45 min, whereas without contrast it could take approximately half the time, leading to shorter times in the scanner. Figure 1 shows some examples of some LGE images.

Machine learning (ML) is a set of techniques in artificial intelligence (AI) which refers to computer algorithms with human-like intelligence developed to accomplish specific tasks. Deep learning (DL) algorithms, which are a set of ML techniques based on neural networks, are useful for medical imaging related tasks such as those involving diagnosis of diseases (3, 4). Such diagnoses often consist of one or a combination of several ML methods involving image segmentation or classification. Image segmentation involves identifying and marking the region of interest (ROI) while image classification involves extracting features from the ROI and uses those features as a basis for classifying patients or diseases. The UNet (5) and the ResNet (6) are examples of popular segmentation and classification algorithms, respectively. The use of ML methods such as support vector machines (SVM) (7, 8) and decision trees (DT) (9) are common in applications involving identification of latent relationships in patient phenotypes (10) and development of predictive models (11, 12). Models based on DTs methods are easy to interpret (i.e., are white box models) while those based on SVM methods are versatile and effective including in high dimensional spaces such as when the number of features is greater than the number of sample points.

In this study, our goal is to investigate and analyze pre- and post-contrast CMR images to predict post-contrast information (i.e., presence, location, and/or extent of MI scar) from pre-contrast information only (i.e., without having to administer contrast to patients) using ML. This subject has recently gained the interest of many researchers (13–21) but despite these interests, many open problems and challenges on this subject still exist. The accurate prediction of contrast information without contrast administration with ML methods is very challenging for many reasons. Qualitatively assessed interpretation by expert humans of the images are recorded in free text and are highly variable, while quantitative ground-truth of these images are not recorded. In addition, format, and quality standardization of CMR imaging data for ML does not exist.

In this article, in order to predict contrast information from non-contrast CMR images, we describe a pipeline for processing routinely acquired pre-contrast cine short-axis (SAX) CMR and post-contrast LGE images so that these images can be used for preparing ground truth for training models that can predict the location of the epicardial and endocardial walls and the location and the extent of the scar. We explore two approaches, namely, segmentation and classification approaches. For the segmentation approach, we use the popular UNet DL model in our attempts to discover the extent and location of the scar given the pre-contrast cine SAX image frames. For the classification approach, we use the ResNet50 classification model in our attempts to distinguish between the ischemic and non-ischemic cases from the non-contrast cine SAX image frames. The performance of the ML algorithms can be significantly improved by extracting features that could be useful or relevant in the model training process for solving particular problems. This feature extraction can be described as a data transformation process (and may or may not require domain knowledge of the problem). Some advantages of the feature extraction process in addition to performance and predictive accuracy improvements includes dimensionality reduction of the feature space, noise reduction, and improvement in the speed of convergence of the learning algorithms (22–24). Thus, in other to use other ML approaches for the classification task, we extract data from the cine SAX images that capture complex patterns representing subtle signal and functional changes in the cine CMR due to myocardial tissue-specific abnormalities and used for qualitative prediction. In particular, we focus on capturing three sets of data from the cine SAX images, namely, the optical flow data, the rate of change of myocardial area, and the radiomics data. Optical flow (image velocity) measurement is a fundamental method in the processing of a sequence of images (successive frames) and its goal is to compute an approximation of a 2D motion field from spatiotemporal data of image intensities (25). Radiomics features are high-dimensional handcrafted quantitative features that are based on mathematical and statistical methods extracted from images (26). They have recently been used on a wide range of problems, such as identifying the causes of myocardial hypertrophy (27) or detecting fibrosis in patients with hypertrophic cardiomyopathy (28). We use the three sets of data to explore two supervised ML methods, namely, the SVM and the DT methods to develop predictive models for classifying pre-contrast cine SAX images as being a case of MI or being free of myocardial scar.

### State of the art

Recently, the attempt to predict post-contrast information without contrast administration is attracting the attention of ML researchers and clinicians alike. Efforts to tackle this challenging task either treats the problem as a pixel-wise tissue identification problem (29) where the extent and location of scar is sought or as an image synthesis problem (14) which involves the generation of images predicting what the post-contrast image would look like.

Changes in mechanical properties of myocardium caused by infarction can lead to regional wall motion abnormalities. This phenomenon inspires the pixel-wise tissue identification approach given in Xu et al. (29) where the proposed DL architecture consists of three connected function layers: the heart localization layers which automatically crop the ROI (i.e., the LV from the cine SAX image frames); the motion feature extraction layers which use long short-term memory (LSTM) recurrent neural networks and optical flow techniques to build local and global motion features through local intensity changes and global intensity changes between adjacent images; and the fully connected layers which learns to predict tissue identities (that is, infarct or not) in each pixel.

In Xu et al. (13), a so-called deep spatiotemporal generative adversarial network (DSTGAN) was used to simultaneously segment and quantify (i.e., infarct size, percentage of infarct size, percentage of segments, perimeter, centroid, major axis length, minor axis length, orientation, and transmurality) MIs directly from the cine MR image. The DSTGAN uses the conditional generative adversarial network (cGAN) DL approach and the input images are cine SAX images. After a network for heart localization process, the DSTGAN technique consists of three components: (i) a multi-level and multi-scale spatiotemporal variation encoder (which actually uses 25 temporal frames from a single slice location), (ii) the top-down and cross-task generator, and (iii) three task labels relatedness discriminators.

In Zhang et al. (14), a cGAN DL model was trained to tackle the challenge using an image synthesis approach. The generator part of the cGAN uses encoder-decoder architecture with cine SAX images, inversion recovery-weighted (IRW) images and T1 mapping images as input to produce virtual native enhancement (VNE) images. The discriminant part of the cGAN uses the VNE images as input and conditioned with LGE images during the model training process. The limitation of this approach is that it requires the acquisition of additional CMR images (IRW and T1 mapping images) that are not (yet) typically acquired in routine CMR imaging in the diagnosis of MI.

Researchers have developed a CNN-based model that identifies ischemic scar slices in computed tomography (CT) angiography of the LV without any contrast agents (15). The model’s algorithm uses LGE images from CMR as ground truth (i.e., a CT-MRI paired dataset) to determine the presence or absence of scar for the binary classification problem. However, this promising approach does not give an idea of the extent or the percentage myocardium affected by scar.

In addition to wall motion abnormalities, myocardial features such as myocardial wall thinning and myocardial lipomatous metaplasia that lead to chemical shift artifacts have been shown to characterize MI (30–32) and suggest that it may be possible for radiomics analysis to identify ischemic scar from non-contrast cine CMR images (16–19). Thus, in Baessler et al. (16), for example, researchers have proposed radiomics texture analysis for the diagnosis of subacute and chronic MI on non-contrast cine CMR images. The approach analyses end-systolic cine SAX images using stepwise dimension reduction (the Boruta feature selection algorithm and the recursive feature elimination method – where a classifier, random forest classifier in this case, is recursively trained and the feature with the smallest ranking score is removed at each iteration), logistic regression ML method and correlation analysis to select features that will enable the classification of end-systolic cine images as cases with or without myocardial scar. The limitation of this approach is that it focuses on texture radiomics features only (i.e., ignoring other radiomics features such as shape) even though features such as wall thinning in myocardial regions are already reported as potential signs of myocardial scar (30). The approach also ignores wall motion abnormalities.

Another radiomics texture analysis approach was proposed in Larroza et al. (17) for differentiating acute MI from chronic MI cases using both contrast LGE images and non-contrast cine CMR images. The approach analyzed LGE images by developing three ML classifiers namely, random forest, SVM with Gaussian kernel, and SVM with polynomial (degree = 3) kernel classifiers. The three counterpart classifiers were developed for non-contrast cine CMR. The recursive feature elimination method with SVM classifier was used as the feature selection technique. Similar to Baessler et al. (16), this approach focused on texture radiomics features only. The major limitation of the texture analysis approaches in Baessler et al. (16) and Larroza et al. (17) is that they have included orientation dependent texture features (obtained from 2D ROI delineation) in their analysis which influence the results if the SAX views are not acquired in a standardized position as presented in those reports.

In Larroza et al. (18), texture analysis was used for classifying myocardial regions of patients suffering from chronic MI into three categories (segments) namely, remote segments (LGE = 0%), viable segments (0 < LGE < 50%), and non-viable segments. LGE CMR images were used to prepare the ground-truth for the non-contrast cine SAX myocardium regions using the 17-segment model. A SVM with radial basis function kernel classifier was trained. Importantly, texture features were calculated in their rotation invariance form in order to evade image rotation as a possible source of bias. Time dimension available in cine sequences are also included as part of analysis in order to take advantage of the information on temporal dimension. The recursive feature elimination method with SVM classifier was used as the feature selection technique. The proposed method also focused on texture radiomics features only.

In Di Noto et al. (19), researchers evaluated radiomics features of LGE regions of CMR images for distinguishing between MI and myocarditis. K-nearest neighbor, linear discriminant analysis (LDA), neural network (multilayer perceptron), SVM, and TreeBagger DT are the five different ML algorithms investigated in the report and the recursive feature elimination method was used as the feature selection technique. However, these analyses were carried out on LGE images and not on non-contrast cine images.

In Avard et al. (20), researchers used radiomics analysis to extract shape, first-order, and texture features for the differentiation of MI and viable tissues (normal) cases in the LV using non-contrast cine CMR images. The whole of the left ventricular myocardium (3D volume) in end-diastolic volume phase was used for the analysis. Ten ML algorithms were investigated for the classification tasks and the SVM and the logistic regression-based models show superior performance compared to other methods on evaluation dataset.

A state-of-the-art review of the methods for delineating LV scar without contrast administration can be found in Wu et al. (21). In general, as far as radiomics analysis is concerned, no specific subset of features has been found to be reliable discriminative of myocardial scar from disease-free regions of the myocardium. Research in this area is still ongoing and progress in this field and progress toward clinical application will require standardization of the discriminative features and evaluation of proposed models to ensure generalizability.

## Materials and methods

### Data acquisition and analysis tools

#### Study population

The study population consists of 272 retrospectively selected CMR studies with diagnoses of MI (*n* = 108) and healthy controls (*n* = 164) from the Barts BioResource between January 2015 to June 2018. Barts BioResource is a local biorepository of Barts Heart Centre (Barts Health NHS Trust, London, United Kingdom) that holds data from prospectively consented (written) patients for cardiovascular research (Ethics REC reference: 14/EE/0007). All images were de-identified prior to analysis.

#### Cardiac magnetic resonance acquisition and myocardial infarction diagnosis

Cardiac magnetic resonance examinations were obtained using 1.5T and 3T scanners (Siemens Healthineers, Germany). The steady-state free precession cine images for SAX were analyzed using CVI42^®^ research prototype software 5.11 built-in ML tool. In order to diagnose for MI, firstly, CMR images – the cine short axis (SAX) images and images of horizontal long-axis (HLA) 4 chamber view and the vertical long-axis (VLA) 2 chamber view – of the patient are taken. The cine SAX images are spatio-temporal, meaning that, for each slice location (in space) of the left ventricle (LV), several images are taken (in time) over the cardiac cycles. Next, gadolinium contrast agent of 0.1 to 0.2 mmol/kg is administered to the patient intravenously. Then, after around 10 min wait, the second set of CMR images are taken using a conventional 2D breath-hold technique. This second set of post-contrast SAX images only have spatial component (have no time component, i.e., one slice for each slice location) and often referred to as the LGE images. It is these LGE images that are scanned for scar in the heart muscle and predominantly of the LV. Both the cine SAX and LGE images cover the whole heart. The diagnosis of MI is made in accordance with the standard definition given in (33, 34).

#### Machine learning software tools

All experiments were conducted on a Nvidia Tesla M40 machine using Python programming language with the following packages: Scikit-learn (Version 0.24.0) (35) was used to implement SVM and DT, Pyradiomics (Version v3.0.1) (36) was used for radiomics feature extraction, TensorFlow 2.0 Python API machine learning framework (Version 2.7.0) (37) was used for implementing UNet and ResNet50 DL architectures, and MATLAB (Version 9.7.0.1586710, R2019b, Update 8) was used for image registration as part of the ground truth data preparation pipeline.

### Segmentation approach

We carry out experiments with DL-based image segmentation and classification architectures, the UNet and the ResNet50, respectively, in order to derive contrast information without contrast administration.

#### Ground truth data pre-processing pipeline

**FIGURE 2:**
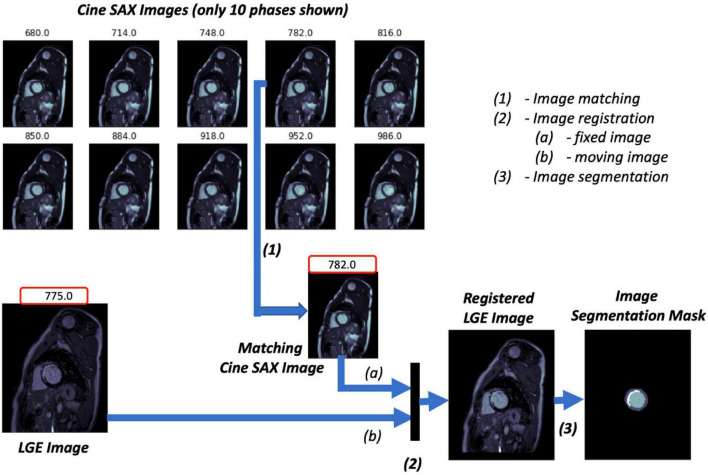
Image ground truth data pre-processing pipeline. The trigger times (TT) in ms for the 10 cine short axis (SAX) images shown are given at the top of each image. The cine SAX frame with closest TT match to the late gadolinium enhancement (LGE) image’s TT is selected (1); this is followed by an image registration process (2), where the “fixed” image and the “moving” image of the registration process are denoted (a) and (b), respectively. The regions of the epicardium, the endocardium, and the scar are segmented in (3).

The three key steps of the image pre-processing pipeline for the ground truth images of the supervised ML problem are illustrated in Figure 2 and described as follows:

1. The pre-contrast cine SAX images and their corresponding post-contrast LGE images for each slice location are extracted. Each cine SAX image is associated with a trigger time (TT), i.e., the slice acquisition time during the cardiac cycle with respect to the peak of the R wave; the peak of the R wave coincides with the early ventricular systole (in Figure 2, for example, the TT in ms for the 10 cine SAX images shown are given at the top of each image). As such the cine SAX image frame of a given slice location whose TT approximately “matches” the TT of the LGE image of the same slice location is selected. The idea is that the heart muscle is at approximately the same position for the two images.
2. The LGE image is then registered with the selected cine SAX image as reference; that is, the LGE image is transformed and resampled into the coordinate system of the cine SAX image (the LGE and cine SAX images are the “moving” and the “fixed” images, respectively, in image registration terminology). The image registration process is an affine transformation consisting of translation, rotation, scale, and shear using the one-plus-one evolutionary algorithm (38) as the optimizer and the Mattes mutual information algorithm (39) as the mutual information metrics (40). MATLAB’s “imregtform” function, for example, can be used to accomplish this image registration process. With image registration completed, the registered LGE image now has the same orientation, scale, and size with its matching cine SAX image. This image registration aims to correct the spatio-temporal misalignment between the pre-contrast matching cine SAX image and the post-contrast LGE image.
3. The registered LGE image is then contoured to mark regions of the epicardium, the endocardium, and the scar. Manual image segmentation was undertaken by trained observers (ER and AK). The LV structures were manually segmented to obtain three labels, namely, the LV cavity (the endocardial wall), the myocardium (the epicardial wall), and the scar. The fourth label (the background) is the non-segmented part of the image.

Given that the patients may have moved (even if slightly) between the pre- and post-contrast image acquisition, the slice location before and after contrast are not exactly the same. A further step of quality control action is taken by removing those slices with significant spatial mismatch between the cine SAX and LGE images. This quality control step was carried out by manual visual inspection. We are then left with 722 cine SAX images and segmentation mask pairs from the 272 subjects. It should be noted that the cine SAX images included images across all slice locations (i.e., all slices between and including the basal and the apical slices).

For the image segmentation task of marking the extent and location of the scar as well as the epicardial and endocardial walls, the model training involves feeding our model with cine SAX images as inputs and their corresponding masked registered LGE images as the ground truth. The model prediction involves feeding the trained model with cine SAX stack as input so that it can predict contoured LGE masks in its output. The segmentation masks are not required for the image classification task, which involves categorizing pre-contrast cine SAX images as cases with MI or non-MI. For any set of cine SAX images, we determined from its corresponding LGE image of the same slice location whether it contained scar or not and then labeled it as such (i.e., Class 0 for non-MI cases, and Class 1 for MI cases). The cine SAX stack for each slice location contained up to 32 image frames (phases) for both segmentation and classification tasks. For the cases with fewer than 32, empty images (zero arrays) were appended with the stack to make 32 frames.

#### UNet segmentation model

**FIGURE 3:**
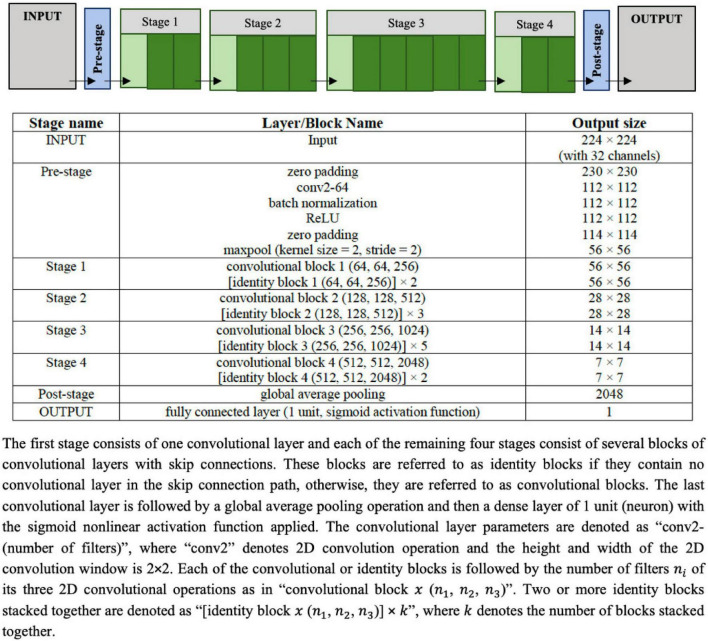
Configuration of the UNet architecture to predict the segmentation mask that marks of the regions of the scar, the myocardium and left ventricle (LV) cavity. The input image is a set of 32 frames of cine SAX image frames. The ground truth (output image) consists of an image with four channels, namely, the background (label = 0), the myocardium (label = 1), the LV cavity (label = 2), and the scar (label = 3).

The UNet architecture given in Figure 3 is used as the image segmentation model, wherein the cine SAX frames are the input, and the ground truth is a segmentation mask that marks the regions of the epicardium, the endocardium and, if present, the scar. The UNet architecture includes batch normalization following the convolutional layers to enhance robustness of the model and drops out 30% hidden neurons in the first three consecutive up-sampling convolutional layers of the architecture to avoid problems associated with model overfitting. The output of the model yields an image segmentation mask with four channels: the background pixels labeled 0, the myocardium labeled l, the LV cavity labeled 2, and the scar labeled 3. The total number of parameters of the model is 75,019,204 out of which 75,011,524 parameters are trainable.

The model training settings are as follows: the 32 input images were resized to 224 × 224. The images were rotated up to ±60° and their intensities normalized as part of the on-the-fly data augmentation. In the normalization of the intensities, similar to the normalization used in Wolterink et al. (41) for the segmentation of cine CMR images, each image has been normalized between (0.0, 1.0) according to the 1 and 99% percentile of intensities in the image. The parameters of the models were randomly initialized, and training proceeded for 100 complete epochs using a batch size of 32 cine SAX images – segmentation mask pairs. The optimization method used was the Adam optimizer, with an initial learning rate of 0.001, decreasing exponentially at a rate of −0.1 after the first 5 epochs. If 30 epochs elapsed with no decrease in the loss function, training was set to cease and the weights from the best epoch is restored as the model’s weights. With the total of 722 cine SAX images and segmentation mask pairs, we have used 70, 15, and 15% as the training, validation, and testing (evaluation) sets (representing, 506, 108, and 108 pairs), respectively.

Channel-weighted (class weighted) dice similarity coefficient (DSC or Dice score) function was used as the loss function. Image segmentation accuracy can be evaluated using DSC, which can be defined in terms of the per pixel classification for the *i*-th channel of a 2D segmentation mask as follows:


(1) $$D\,S\,C_{i}=\frac{2\,\sum_{n=1}^{N}y_{i_{n}}\,{\hat{y}}_{i_{n}}}{\sum_{n=1}^{N}y_{i_{n}}+\sum_{n=1}^{N}{\hat{y}}_{i_{n}}}$$

where *y*_*i_n_*_ and ${\hat{y}}_{i_{n}}$ are the ground truth mask and the predicted mask (the posterior probability obtained after the application of the “softmax” activation function on the output layer of the model), respectively, and *N* is the number of pixels in the mask (224 × 224). The dice loss of the *i*-th channel, 1−*DSC*_*i*_, can be written as:


(2) $$l_{i}=1-\frac{2\,\sum_{n=1}^{N}y_{i_{n}}\,{\hat{y}}_{i_{n}}}{\sum_{n=1}^{N}y_{i_{n}}+\sum_{n=1}^{N}{\hat{y}}_{i_{n}}}$$

The channel-weighted dice loss function *L* for the model can then be written for the 4 channels as follows:


(3) $$L=\sum_{i=1}^{4}\beta_{i}\,l_{i}$$

where β_*i*_ is the associated with channel *i*. In our case, we defined β_1_=0.15, β_2_=0.25, β_3_=0.25 and β_4_=0.35; meaning that, we have assigned more weight to the scar channel than the others, and the background channel has the least weight.

### Classification approach

#### ResNet50 classification model

**FIGURE 4:**
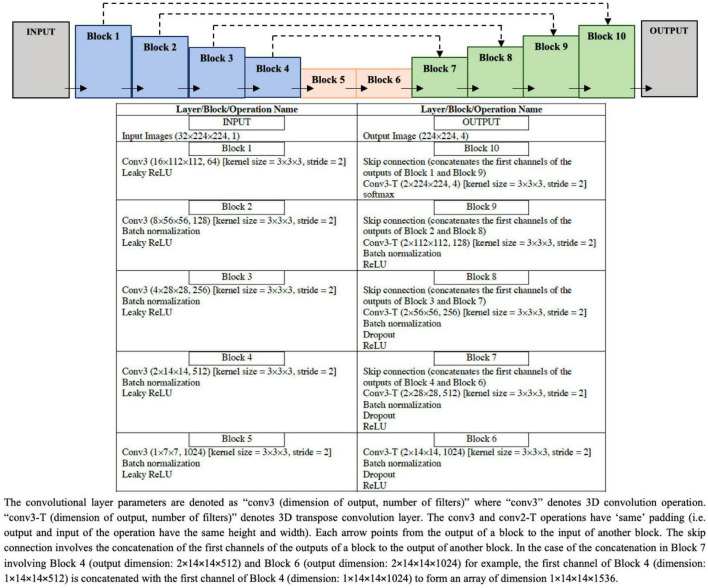
Configuration of the ResNet50 architecture to predict the binary outcome of presence or absence of myocardial scar using the set of 32 cine short axis image frames as input to the model.

The architecture of ResNet50 given in Figure 4 is used to train the DL classification model. Given cine SAX input frames, the ResNet model predicts whether or not the corresponding post-contrast LGE image would contain a scar as a result of MI. The main characteristic of this architecture of ResNet50 is that the number of channels of the input is 32 (i.e., 32 cine SAX images) – as against the 3 channels for a colored RGB image in a standard ResNet50 model. The output of the model is a binary prediction of whether the cine SAX images in a case without MI (class 0) or with MI (class 1) (without or with scar, respectively). The total number of parameters of the model is 23,680,705 out of which 23,627,585 parameters are trainable.

For the model training, the optimization method used was the Root Mean Squared Propagation (RMSProp) optimizer, with an initial learning rate of 0.0001. All other model training settings are the same as those of the segmentation model given in the preceding sub-section.

Of the 506 pairs that constitute the training set, the number of pairs that represent scar cases and cases with no scar are 336 and 170, respectively. In order to address this data imbalance, we used a weighted loss function. Let {(*x*_1_,*y*_1_),(*x*_2_,*y*_2_),(*x*_*n*_,*y*_*n*_),(*x*_*N*_,*y*_*N*_)} denote a training set of *N* samples where *x* is the cine SAX input images and *y* ∈ {0,1}^*C*^ denote a binary one-hot encoded label with *C* 2 in our case, then the weighted loss function is defined as:


(4) $$E_{w}\left(\theta \right)=-\frac{1}{N}[\lambda_{0}\sum_{n=1}^{N}{𝕋}_{0}\left(x_{n}\right)y_{n}\log\left({\hat{y}}_{n}\left(x_{n},\theta \right)\right)+$$

$$\lambda_{1}\sum_{n=1}^{N}{𝕋}_{1}\left(x_{n}\right)y_{n}\log\left({\hat{y}}_{n}\left(x_{n},\theta \right)\right)]$$

where θ denotes the trainable parameters of the model; ${\hat{y}}_{n}\,\left(x_{n},\theta \right)$ is the posterior probability obtained after the application of sigmoid activation function on the output layer of the model; 𝕋_0_(*x*_*n*_) and 𝕋_1_(*x*_*n*_) are functions that indicate whether image *x_i_* belongs to class 0 (cases with no scar) or class 1 (cases with scar), respectively; and λ_0_ and λ_1_ are weights that penalize the loss function for false negative errors and false positive errors, respectively. The weights, λ_0_ and λ_1_, can be computed using the following equation:


(5) $$\lambda_{i}=\frac{1}{k_{i}}\cdot \frac{N}{c}$$

where *k_i_* is the number of samples belonging to class *i*. In our case, *N*=506; class 0 and class 1 are subgroups indicating the collection of samples with no scar and with scar, respectively; then, λ_0_=(1/336)×(506/2)=0.753 and λ_1_=(1/170)×(506/2)=1.488. In other words, the images representing scar cases (class 1) are weighted as being more valuable than those representing no scar cases (class 0).

#### Feature extraction

In order to use ML methods for identifying MI using pre-contrast cine SAX images, we explore the data-driven approach by capturing three sets of data from the cine SAX images, namely, the optical flow data, the rate of change of myocardial area, and the radiomics data.

##### Optical flow data

The goal of optical flow is to compute an approximation of 2D motion field from spatiotemporal data of image intensities (25). Using Lucas–Kanade method (see Supplementary Material) for estimating optical flow velocities, we have chosen the window size (spatial neighborhood Ω) of 8×8 and selected a Gaussian filter *w* of kernel size 5×5 with a SD of 3 along each of *x* and *y* directions (σ_*x*_=σ_*y*_=3.0). The magnitude of the optical flow velocities *v*=|**v**| can be computed as follows:


(6) $$v=\sqrt{v_{x}+v_{y}}$$

*v_x_* and *v_y_* represent *x* and *y* component of *v*. The magnitude of the displacement of the optical flow field *r* (pixel-wise displacement) can therefore be computed as follows:


(7) r=v⁢Δ⁢t

where Δ*t* is the time difference between acquisition of the two successive images. We further reduced the dimension of the displacement matrix *r* using principal component analysis (PCA) approach and vectorized (reshaped) the resulting matrix into a row vector.

**FIGURE 5:**
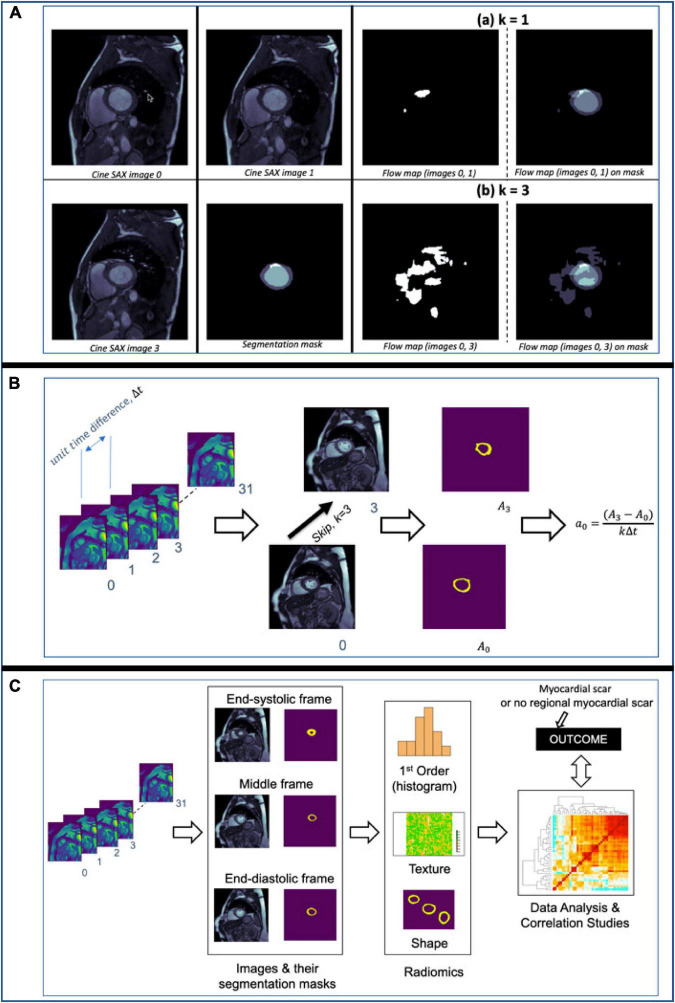
**(A)** Flow maps showing the pixels that have been displaced between the time the two cine SAX image frames were acquired with the skip intervals *k*=1 in (a) and *k*=3 in (b). On the left in (a), flow map (images 0, 1) represents the pixel-wise displacement between image 0 and image 1, and the corresponding the pixel-wise displacement between image 0 and image 3 is shown at the bottom. The images on the left show the super-imposition of the flow map images on the segmentation masks. **(B)** An illustration of the computation of rate of change of myocardial cross-section area for a given slice location. The rate of change of myocardial area between frames captures the pixel-wise area change information of slices through the cardiac cycle. **(C)** An illustration of the computation of radiomics features of three frames namely, the end-diastolic (ED) frame, end-systolic (ES) frame and the “middle” frame in between the ED and ES frames. The shape, first-order, and texture radiomics features are extracted and then a statistical test (Pearson correlation analysis) to assess the significance of these features in relation to the binary outcome (i.e., with or without MI).

For illustration, Figure 5A shows flow maps which helps to visualize the pixel-wise displacement between two cine SAX image frames images 0 and 1 in (a) and images 0 and 3 in (b). We refer to the interval between image frames as the “skip” interval, *k*; in (a), the *k*=1 and in (b), the *k*=3. The images on the right show the super-imposition of the flow map images on the segmentation masks. Equation 7 assumes *k*=1 (i.e., the two image frames are next to each other, e.g., the 2nd and 3rd frames, in a cine SAX set of 32 frames). The choice of *k*=1 reduces the number of displacement matrices. In the case of 32 cine SAX frames for a given slice location, the choice *k*=3 results in having 11 displacement matrices where the magnitude of the displacement is *r*=*v*×*k*Δ*t* and Δ*t* can be calculated by subtracting the TT as follows: Δ*t*=*t*_*i* + 1_−*t*_*i*_ where *t_i_* is the TT associated with image *i*.

##### Rate of myocardial area change

The rate of change of myocardial cross-section area between successive frames of a given slice location, *a_i_*, captures the pixel-wise area change information of slices through the cardiac cycle and can be expressed as follows:


(8) $$a_{i}=\frac{△\,A}{k\,△\,t}$$

where Δ*A*=*A*_*i* + *k*_−*A*_*i*_; *A*_*i*_ and *A*_*i+k*_ are the areas of myocardium for the *i*-th and (*i+k*)-th image frames at a given slice location, respectively, and *k* is the skip interval. Figure 5B illustrates the computation of *a_i_* for a given slice location. Thus, for the up to 32 cine SAX frames for a given slice location and with *k*=3, we compute 11 values of *a*_*i*_ (i.e., *a*_*i*_ for *i*=0, 2, 10).

##### Data from radiomics

Rather than computing the radiomics features of each of the 32 cine SAX frames, we extracted radiomics from three frames: end-diastole, end-systole and the “middle” frame, which is precisely in between the end-diastolic and end-systolic frames. We extracted 306 shape, first-order, and texture radiomics features for the three frames of interest using the Pyradiomics open-source package. It should be noted that only the myocardium is segmented (i.e., an image segmentation mask with the background pixels labeled 0, and the myocardium labeled l). More details on radiomics features can be found in Freeman et al. (42) and Chu et al. (43).

The pixel spacing varies from 1.41 to 2.34 mm and to correct for differences in pixel size, each of the 2D image slices were resampled to 1.9 mm × 1.9 mm spacing through a one-dimensional (1D) area interpolation. Similar to Di Noto et al. (19), owing to strongly anisotropic CMR acquisition (i.e., out-of-plane information is intrinsically poorer), we resampled on the XY plane to preserve in-plane information. Furthermore, to account for sensitivity of radiomics features to intensity variation associated with the image acquisition process, intensity normalization of the images is carried out prior to the extraction of radiomics features. For the intensity normalization, the 1–99% intensity normalization (i.e., 1–99% percentile of intensities) with 256 intensity levels of each image has been used. In our pre-processing step, we have not performed bias correction although it may improve the inhomogeneity of images (44).

From the 306 set of radiomics features, a subset of features that are highly correlated (i.e., redundant features) are removed. In particular, features with Pearson correlation coefficient higher than 0.9 are removed while retained only one of those correlated features, resulting in 144 features (48 features for each of the three frames). We have chosen this value (*r* > 0.9), similar to Rauseo et al. (45), to ensure only highly correlated features are removed. We then carried out statistical test to assess the significance of the radiomics features in relation to the outcome – in this case, a binary outcome of whether the cine SAX images results predict MI or not. A *p* < 0.001 was considered to be statistically significant, leading to the selection of 38 radiomics features (15 systolic frame, 9 diastolic frame, and 14 middle frame features). Figure 5C illustrates the computation of radiomics features as we have described here.

#### Support vector machines and decision tree machine learning methods

Given that we now have information on the optical flow data, data on rate of change of myocardial cross-section area at any given slice location, and the radiomics data, we explored two supervised learning methods to qualitatively predict the presence/absence of scar, namely, the SVM and the DT methods.

Firstly, we carried out the *z*-score standardization. This standardization method transforms the feature space to have zero mean and unit variance, and has been shown to improve speed of convergence of SVM algorithms for classification problems and SVM model performance (46). Normalization is important for SVM method (47, 48) [in fact, SVM method may not be appropriate for some problems without normalization (49)]. While feature space transformation using standardization or normalization plays an important role in Euclidean distance minimization-based algorithms (e.g., SVM, neural networks, K-nearest neighbor, etc.), algorithms that are insensitive to feature scaling (variance scaling), such as DT, are not affected by the transformation (50).

Next, we split the dataset into random 80% train and 20% test subsets (representing, 577 and 145 sets, respectively). The training set was used for training SVM and DT models and the test set was used for unbiased evaluation of these models. Further details of the SVM with the radial basis function kernel (7, 8) and DT (9) methods used in this work are provided in the Supplementary Material.

### Model evaluation methods

The DSC is a measure of similarity between the label and predicted segmentation masks and is often used to evaluate performance of ML segmentation models. Given two sets (two images in this case) *A* and *B*, DSC score can be expressed as follows:


(9) $$D\,S\,C=\frac{2\,\left|A\cap B\right|}{\left(\left|A\right|+\left|B\right|\right)}$$

where |*A*| and |*B*| represent the cardinalities of set *A* and *B* (i.e., the number of elements in each set), respectively. The DSC, which has a range of [0,1], is a useful summary measure of spatial overlap that can be applied to quantify the accuracy in image segmentation tasks. Computing the DSC of several images from a segmentation model and evaluating the mean DSC (or other statistical validation metric) allows the comparison of the model with other models.

The performance of a classification models can be evaluated using the following metrics: confusion matrix, F1 score and accuracy score. The confusion matrix is a table that describes the performance of a model on a set of data for which the true labels are known by summarizing the count values for each class. For binary classification, the confusion matrix counts the number of true negative (*TN*), false negative (*FN*), true positive (*TP*) and false positive (*FP*). Precision (model’s ability not to misclassify a negative sample as positive, i.e., a measure of result relevance), recall (model’s ability to find all positive samples), the F1 score (the harmonic mean of the precision and recall) and the accuracy score (the fraction of the correct prediction out of the total number of samples) are other performance metrics useful in evaluating binary classification tasks.

## Results

### Segmentation approach

**FIGURE 6:**
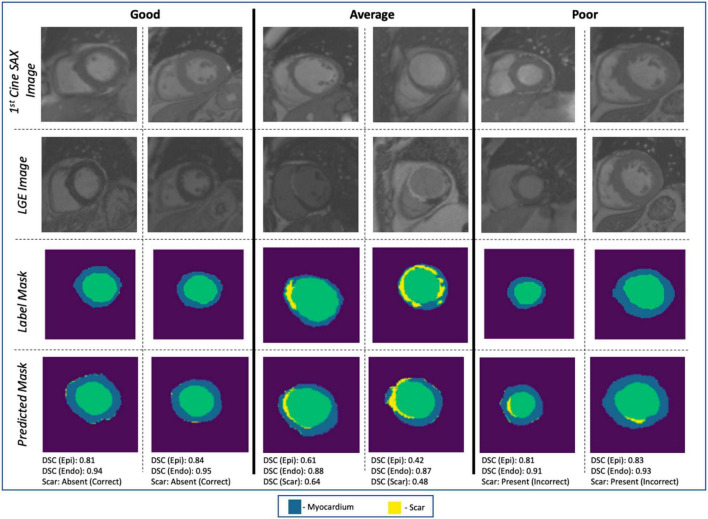
Examples of image segmentation results. The “Good” (correctly identifying the absence of myocardial scar due to MI), “Average” [correctly identifying the presence of the scar with *DSC* ∈ (0.4, 0.65)], and “Poor” (incorrect identification of the presence of the scar). Cases with *DSC* < 0.4 are not shown in the figure.

**FIGURE 7:**
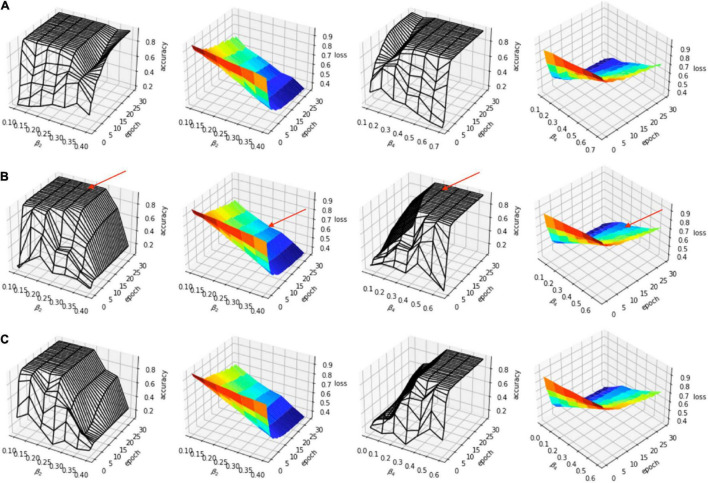
Simulation experiment for **(A)** β_1_=0.1, **(B)** β_1_=0.15, and **(C)** β_1_=0.2. In each case, β_2_=β_3_. The arrows in **(B)** indicate the choice (β_1_=0.15, β_2_=β_3_=0.25, β_4_=0.35).

The evaluation of the predicted results of the UNet segmentation model was performed using the DSC score as the performance metric. For the testing set (*n* = 108), the mean DSC is 0.20 [±0.17 SD; 0.64 maximum; 0.14 median (50% percentiles)] for scar, the mean DSC is 0.51 (±0.21 SD; 0.86 maximum; 0.52 median) for epicardium (the myocardium), and the mean DSC is 0.75 (±0.20 SD; 0.94 maximum; 0.85 median) for endocardium (the LV cavity). These results are summarized in Table 1. The results from fivefold cross-validation are presented in Table 2 and give the mean (±SD) DSC as 0.24 (±0.12), 0.48 (±0.23), and 0.77 (±0.18) for the scar, epicardium, and endocardium, respectively. In general, we observe that the UNet model is able to discover the regions of the endocardium and the epicardium with high degree of accuracy. The accuracy of the prediction of the extent and location of the scar is much lower. Some examples of the results of the UNet segmentation model are presented in Figure 6. Only the first cine SAX image in the set of 32 cine SAX images is shown in each example. In relation to the channel-weighted dice loss function, although the choices of β_*i*_ were empirical, our experiments as given in Figure 7 shows that our choice of these values are reasonable. In Figure 7, we have presented the results of our simulation experiment for the first 30 epochs to show the accuracy (pixel-wise categorical accuracy) and loss (Equation 3). The arrows in (b) indicate our choice and both the loss and accuracy are satisfactory compared to other possible choices. For this experiment, we have only considered the cases of (A) β_1_=0.1, (B) β_1_=0.15, and (C) β=10.2.

**TABLE 1:** The mean, maximum and median Dice scores of the UNet segmentation model.

	Mean (SD)	Maximum	Median
Scar	0.20 (±0.17)	0.64	0.14
Epicardium	0.51 (±0.21)	0.86	0.52
Endocardium	0.75 (±0.20)	0.94	0.85

**TABLE 2:** Comparing the mean, maximum, and median Dice scores of five UNet segmentation models calculated from fivefold cross-validation.

	Mean (SD)	Maximum	Median
**Model 1**			
Scar	0.16 (±0.14)	0.44	0.12
Epicardium	0.48 (±0.23)	0.86	0.54
Endocardium	0.74 (±0.23)	0.95	0.83
**Model 2**			
Scar	0.17 (±0.13)	0.42	0.13
Epicardium	0.41 (±0.27)	0.90	0.46
Endocardium	0.67 (±0.29)	0.97	0.81
**Model 3**			
Scar	0.24 (±0.15)	0.53	0.27
Epicardium	0.46 (±0.24)	0.87	0.48
Endocardium	0.77 (±0.18)	0.97	0.83
**Model 4**			
Scar	0.20 (±0.17)	0.64	0.16
Epicardium	0.44 (±0.25)	0.88	0.48
Endocardium	0.72 (±0.22)	0.96	0.80
**Model 5**			
Scar	0.20 (±0.12)	0.42	0.14
Epicardium	0.41 (±0.25)	0.87	0.43
Endocardium	0.72 (±0.22)	0.96	0.79

### Classification approach

**FIGURE 8:**
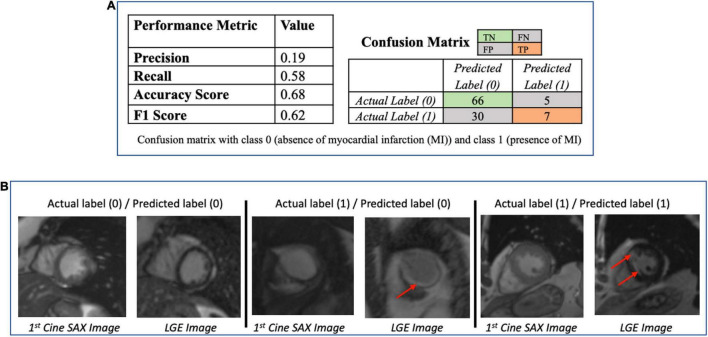
**(A)** Performance of the ResNet50 classification model (to determine presence/absence of myocardial scar) using the test dataset (*N* = 108). The low value of the precision score (0.19) makes the model unsatisfactory in determining the presence of absence of myocardial infarction. **(B)** Some examples of the prediction of the ResNet50 classification model. The red arrows point to the location of the scar. The middle column shows the case that the classification models got wrong.

The performance metrics (confusion matrix, precision, recall, accuracy, and F1 scores) of the trained ResNet50 model on the evaluation dataset are given in Figure 8A. Figure 8B provides some examples of the predictions of the classification models. The precision score (0.19) is particularly poor for ResNet50 model (i.e., the number of TP of the confusion matrix is relatively small) making the model unsatisfactory in determining the presence of absence of MI.

**FIGURE 9:**
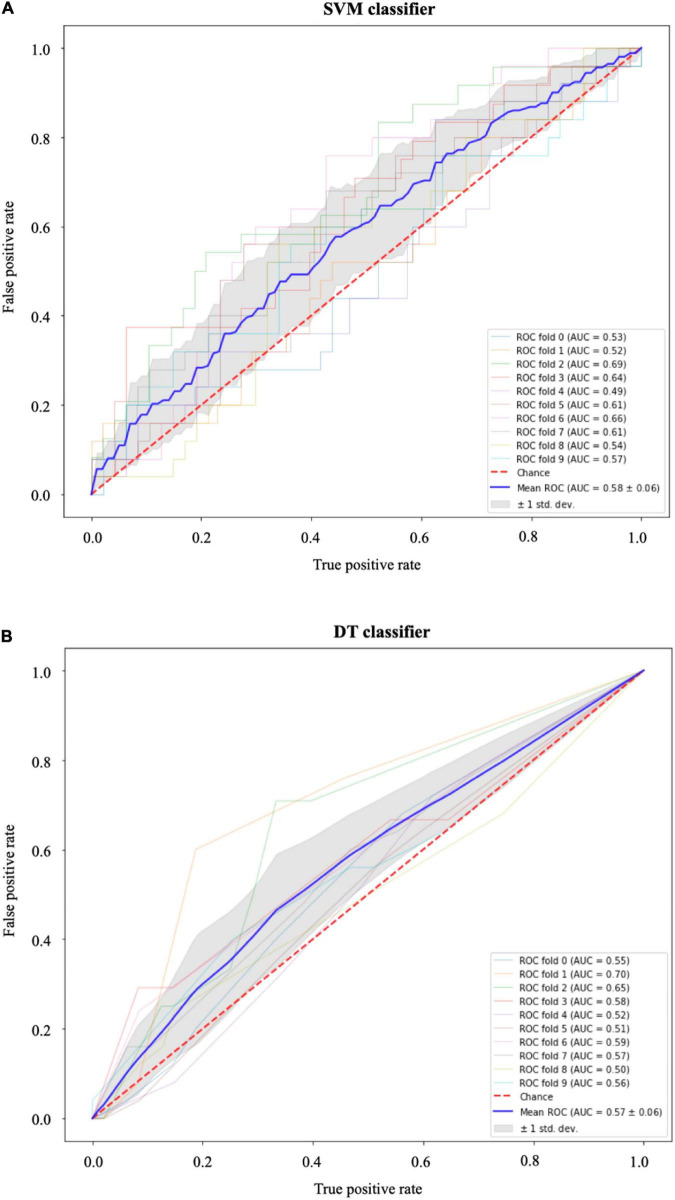
Receiver operating characteristic (ROC) curves for **(A)** the support vector machines (SVM) and **(B)** the decision tree (DT) classifiers. In both models, the area under the curve (AUC) values (0.5 < AUC < 1) show that the classifiers have some predictive power above “chance” to distinguish between the positive class values from the negative class values. The gray area indicates ±1 SD calculated from 10-fold cross-validation.

The results of the SVM with radial basis function kernel are shown in (a) of Table 3 with different combinations of data, namely, (i) the optical flow “plus” rate of myocardial area change data ($\frac{△\,A}{k\,△\,t}$); (ii) the optical flow “plus” $\frac{△\,A}{k\,△\,t}$ “plus” radiomics data; and (iii) $\frac{△\,A}{k\,△\,t}$ “plus” radiomics data. We observe improvement in prediction accuracy of the SVM model from the confusion matrices moving from left to right; that is, the model with the rate of myocardial area change data “plus” radiomics data has the highest accuracy and F1 scores of 0.68 and 0.69, respectively. The results of the DT model are shown in (b) of Table 3. Similarly, DT model where the input consists of the three combination of data features has the best performance in terms of the precision score. Figure 9 shows the receiver operating characteristic curves (ROC) for the best performing SVM and DT classifiers calculated from 10-fold cross-validation. The area under the curve (AUC) values (0.5 < AUC < 1), that is, 0.58 ± 0.06 and 0.57 ± 0.06 for SVM and DT classifiers, respectively, show that the classifiers have some predictive power to distinguish between the positive class values from the negative class values.

**TABLE 3:** Performance of machine learning data-driven approaches, with methods (a) support vector machines (SVM) using radial basis function (RBF) kernel and (b) decision tree, for different combinations of the optical flow, rate of change of myocardial area and radiomics data.The SVM model had the best performance in terms of accuracy and F1 scores when the input consists of the rate of change of myocardial area and the radiomics data. The DT model had the best in terms of the precision score when the input consists of the optical flow, the rate of change of myocardial area and the radiomics data.

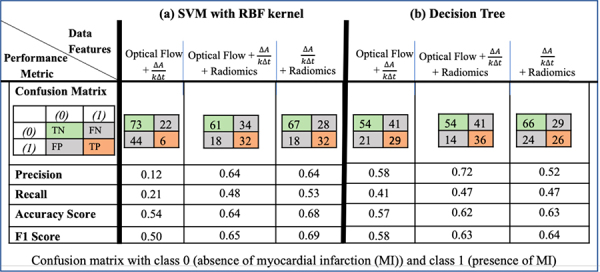

**FIGURE 10:**
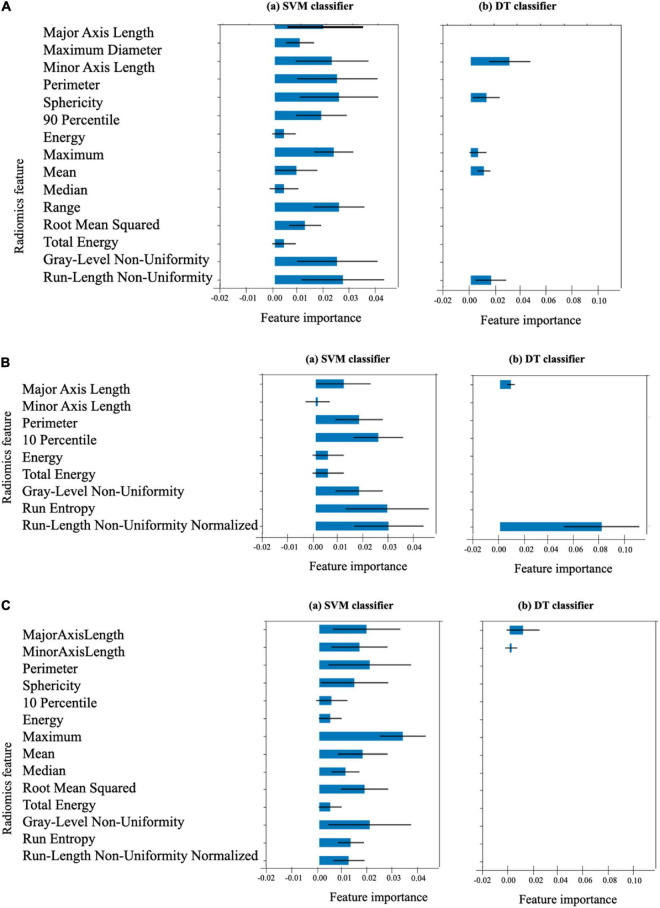
Importance of the 38 radiomics features associated with **(A)** the end-systolic frame (15 features), **(B)** the end-diastolic frame (9 features), and **(C)** the middle frame (14 features) for the SVM and DT models. The length of the blue bar indicates the importance of the feature to the generalization power of the model (the black line is the ±SD). The DT classifier considers only the 9 features indicated as the only important features for its own classification.

The list of 38 radiomics features (15 systolic frame, 9 diastolic frame, and 14 middle frame features) that are considered statistically significant (*p* < 0.001) are given in Table 4. The definition of these feature can be found in (36). In order to estimate the importance of each radiomics feature to the SVM and DT models, permutation feature importance method (51) is used. This involves shuffling each of the features *N* number of times (*N* = 10 in our case) and estimate the importance of the feature by measuring the decrease in model predictive accuracy. Figure 10 shows the importance of each radiomics feature for the (optical flow “plus” $\frac{△\,A}{k\,△\,t}$ “plus” radiomics data) SVM and DT models computed using the test data set (i.e., using the training set data may indicate features that are important during model training only and these may not generalize).

**TABLE 4:** Statistically significant radiomics features of the three cine short-axis image frames.

Systolic frame	Diastolic frame	Middle frame
2D shape-based features: •Major axis length •Maximum diameter •Minor axis length •Perimeter •Sphericity	2D shape-based features: •Major axis length •Minor axis length •Perimeter	2D shape-based features: Major axis length •Minor axis length •Perimeter •Sphericity
First-order statistics features: • 90th percentile • Energy • Maximum • Mean • Median • Range • Root mean squared •Total energy	First-order statistics features: •10th percentile •Energy •Total energy	First-order statistics features: •10th percentile •Energy •Maximum •Mean •Median •Root mean squared •Total energy
Gray level run length matrix Texture-based features: •Gray level non-uniformity •Run length non-uniformity	Gray level run length matrix Texture-based features: •Gray level non-uniformity •Run entropy •Run length non-uniformity normalized	Gray level run length matrix Texture-based features: •Gray level non-uniformity •Run entropy •Run length non-uniformity normalized

## Discussion

### Summary of findings

In this study, we have presented a novel pipeline for processing routinely acquired CMR images that can be used as ground truth images for supervised and unsupervised ML methods in order to predict presence, location and extent of MI from contrast agent free cine SAX set.

For the UNet segmentation model whose input are the cine SAX frames and whose output is the segmentation mask that marks the regions of the epicardium, endocardium and the scar, the overall performance based on the average DSC score for the 108 test set is 0.75 (±0.20) for the endocardium, 0.51 (±0.21) for the epicardium and 0.20 (±0.17) for the scar; and 0.24 (±0.12), 0.48 (±0.23), and 0.77 (±0.18) for the scar, epicardium and endocardium, respectively, from fivefold cross-validation. At first glance, the prediction accuracies of the epicardium or endocardium appear low given that medical image segmentation is a widely studied subject (3) and the state-of-the-art average DSC could reach 0.94 for CMR (52) or 0.885 for cardiac CT images (53). We note that in those previous studies, the models (i.e., the one-input and one-output models) involved a single image and the prediction of the models is compared with the ground truth obtained after segmenting the input image. In our case (i.e., a 32-inputs and one-output model), the model input is a set of 32 image frames and the DSC score was obtained for only one of these images whose TT “matches” the TT of the LGE image of the same slice location; thus, comparing the predicted mask with only one mask (i.e., mask of the registered LGE image only out of the 32 possible masks) does not necessarily paint the overall picture of the level of the accuracy (and therefore, cannot be benchmarked with standard image segmentation models involving one-input, one-output models).

Also, in the LGE image registration for data pre-processing, we have used affine image transformation. Other non-rigid image registration methods, e.g., the free-form deformation (FFD) image registration methods (54, 55), may be more suitable for recovering motion and deformation since they can capture local motion of the myocardium into the registration process. Using FFD-based methods for spatio-temporal CMR image registration to correct spatial misalignment caused by patient motion and temporal misalignment caused by the motion of the heart may improve the accuracy of registration and, consequently, the manual segmentation for the ground truth and the prediction of the UNet model. Such methods may therefore be considered in a future work on this subject.

Moreover, the limited amount of dataset (the training, validation, and testing sets representing, 506, 108, and 108 cine SAX images – mask pairs, respectively) could explain this relatively low level of prediction accuracy for the scar (which is a more difficult problem even for human experts at times). Importantly, we note that we have used the channel-weighted DSC function as the loss function and have assigned 0.15, 0.25, 0.25, and 0.35 as weights of the background, epicardium, endocardium, and scar (i.e., assigning more importance to the scar channel than others). Future work on this project will involve experimenting with a much larger dataset as well as exploring different choices of weights assigned to the channels. It is worth mentioning that none of the other loss functions we have experimented so far [including the sparse categorical cross entropy loss and (unweighted) dice loss] were able to discover the scar at all (despite some varying degree of successes in their discoveries of the epicardium and endocardium).

The performance metrics of ResNet classification model that predicts whether a cine SAX image frames constitute an MI case or non-MI case show that the model’s performance is poor (see Figure 8). Of particular note here is the precision score ($\frac{7}{37}$, i.e., 0.19 – see also the confusion matrix). ResNet is a very successful DL architecture (3, 53) and the low performance of our ResNet model in this case (which could be for several reasons, e.g., not enough dataset or not information from the given dataset that will enable the model learn the underlying model parameters) emphasize difficulty and complexity of the problem we intend to solve. The lack of sufficient information could also be as a result of reduction of the resolution of the cine SAX images due to resizing (i.e., 224 × 224) although resizing can sometimes be necessary due to hardware limitations or to ensure all input images have common size. This motivated the data-driven ML approaches involving the use of the SVM and DT methods. In using these methods, we have experimented with the use of a combination of three sets of model input data that were captured from the cine SAX images, namely, the optical flow data, the rate of change of myocardial area, and the radiomics data. The SVM method had the best performance of accuracy score and F1 score of 0.68 and 0.69, respectively, when we included data from rate of change of myocardial area and the radiomics as input. The precision score was 0.64. The DT method had the best performance in terms of precision reaching 0.72 when the three sets of data are combined as input. In this case, the accuracy score and F1 score are 0.62 and 0.63, respectively. The best performing models of the data-driven ML methods outperforms the ResNet model on the precision score metric. Also, the SVM and DT models’ AUC values (0.5 < AUC < 1) show that these classifiers do indeed have predictive power above “chance” to distinguish between the positive class values from the negative class values.

### Related work

The pixel-wise tissue identification approach given in Xu et al. (29) is an interesting approach given that it does not require any preliminary segmentation of myocardial walls, captures the dense motion of the myocardium and integrates both local and global motion features for its prediction. The main problem with the approach however is the absence of the ground truth data preparation pipeline or any technique necessary to address the spatio-temporal misalignment between the pre-contrast and post-contrast image. Given the complex nature of this problem and the reported accuracy of 95.03% mean Dice score of the trained model for the identification and segmentation of the scar from cine SAX images, it is most likely that the model – trained on the dataset of 165 cine CMR patients (140 diagnosed with MI and 25 control cases) – suffers from overfitting issues. Moreover, even simpler DL-based image segmentation problems involving CMR images hardly achieved this level of accuracy to date [for example, in Bai et al. (52), endocardium and epicardium segmentation models achieved 0.88 (0.03) and 0.94 (0.04) mean (±SD) Dice scores, respectively; in Jacobs et al. (56), myocardial segmentation model achieved 0.86 (±0.06) Dice score on gadolinium-enhanced CMR images; and in Zhuang et al. (57), myocardial segmentation of the mid-ventricular slice achieved 0.86 (±0.07) inter-observer Dice score on LGE CMR images]. It should be noted that in Zhang et al. (58), researchers have used the framework given in Xu et al. (29) (i.e., with the following main components: LV localization; the motion feature extraction layers which use LSTM and optical flow techniques; and the fully connected layers) for a training dataset that consists of only chronic MI (*n* = 169) and control (*n* = 69) patients. The Dice score of 86.1% (±5.7) was reported in the study but this was the result of a small test set [chronic MI (*n* = 43) and control (*n* = 18) patients] from a single vendor and single center (i.e., the same vendor and center as the training set). The approach and dataset presented in this article are not limited to chronic MI cases only.

The DSTGAN approach given in Xu et al. (13) uses a total of 495 cine SAX images and segmentation mask pairs (i.e., 25 cine frames for each segmentation mask) from 165 patients (140 acute MI patients and 25 non-MI patients), the approach demonstrates impressive performance of 96.98% pixel-wise classification on a 10-fold cross-validation test (i.e., the test set consists of approximately 49 cine SAX and segmentation mask pairs).

The cGAN model proposed in Zhang et al. (14) was trained and tested on a dataset of 2,695 and 345 triplets, respectively. The performance of the model (for *n* = 326 datasets) measured by the correlation with LGE images are [*r* 0.77–0.79; intraclass correlation coefficients (ICC) = 0.77–0.87; *p* 0.001] in detecting and quantifying hyperintensity myocardial lesions and (*r* 0.70–0.76; ICC = 0.82–0.85; *p* 0.001) in detecting and quantifying intermediate-intensity lesions. Moreover, as the authors rightly mentioned, this approach is relevant in the image acquisition stage, meaning that, clinicians will still have to visually scrutinize each of the synthesized images for the location and extent of scar in order to diagnose MI. Importantly, the T1 mapping images contain information about the characteristics of the tissues, and MI scar can somewhat be visible in these images – for example, refer to Figure 4 in the original article. Thus, in our view, the problem solved in Zhang et al. (14) seems to take a relatively complex approach since the task can be reduced to a simpler image synthesis or segmentation task (i.e., synthesize a postcontrast image or obtaining an image segmentation mask from a T1 mapping image). Moreover, as highlighted in Manisty et al. (59), T1 mapping images and LGE images are not imaging equivalent (i.e., interchangeable) myocardial disease processes, so one cannot be expected to replace the other. The approach proposed in this article differs to this model as its outcome is to diagnose with minimal amount of imaging data (cine SAX images only) as input and determine whether it is a case of infarction or not.

In the CNN-based model developed in (15) that identifies ischemic scar slices in CT angiography of the LV, with CT images as input and a training set of 200 patients of which 83 are with scar, the trained network achieved an accuracy of 88.3% on a 10-fold cross-validation metric.

The texture analysis approach proposed in (16) uses end-systolic cine SAX images from 120 MI patients [72 large transmural (>20%) patients and 48 small subacute or chronic (≤20% transmural) patients] and 60 control subjects and, using 5 textural radiomics features, reported a 10-fold cross-validation estimate of accuracy of 0.81 for patients with large myocardial scar versus control subjects, and a cross-validation estimate of accuracy of 0.75 for patients with small myocardial scar versus control subjects.

Similarly, the texture analysis approach proposed in Larroza et al. (17) uses LGE images and end-diastolic cine SAX images from 44 MI patients (22 acute MI patients and 22 chronic MI patients) and reported a fivefold cross-validation results. The SVM with polynomial kernel yielded the best classification performance with ROC providing AUC (mean ± SD) of 0.86 (±0.06) on LGE MRI using 72 textural radiomics features. For the cine CMR images, the SVM with polynomial kernel classifier’s performance given by the AUC of ROC of 0.82 (±0.06) from 75 textural radiomics features.

In Larroza et al. (18) where the texture analysis was used to classify myocardial regions of chronic MI patients into remote, viable and non-viable segments, the approach uses end-diastolic cine SAX images from 50 chronic MI patients [randomly split into training (30 patients) and testing (20 patients) sets] and, using 5 textural radiomics features and a fivefold cross-validation, reported AUC under ROC of 0.849 with sensitivities of 85, 72, and 92% for remote, viable, and non-viable segments, respectively.

In Di Noto et al. (19), radiomics features where captured from LGE images in order to classify the images from 173 patients (111 with MI and 62 with myocarditis) into MI and myocarditis. The approach involved both 2D and 3D texture analysis to capture textural radiomics features; thus, the proposed method used shape and first-order features in addition to texture radiomics features. Five different ML algorithms were investigated and a stratified 10-fold cross-validation was performed. The SVM classifier achieved the best results (accuracy: 88%) for the 2D features and LDA showed the highest accuracy (85%) for 3D features. In comparison with subjective visual analyses by readers with different experience levels, the radiomics approach was superior to the less experienced reader but performed lower with the experienced reader.

Radiomics analysis was used in Avard et al. (20) to classify MI from healthy patients using a dataset of 50 MI and 20 healthy control cases, the average of univariate AUCs was 0.62 ± 0.08. For multivariate analysis, logistic regression (AUC = 0.93 ± 0.03) and SVM (AUC = 0.92 ± 0.05) yielded optimal performance. It is clear that this is a small and an imbalanced dataset (i.e., MI cases are 2.5 times the number of healthy cases) – and this can have significant influence on the predictive power of the models (classifiers are generally not robust to the change of training data size (60). In their report, the researchers have not mentioned how they have eliminated the impact of class imbalance or how its effect on their results.

Thus, while eliminating the need for contrast will save both time and cost of cardiovascular healthcare and improve the patient experience, accurate prediction of contrast information without contrast administration is a very challenging task. We have presented some promising approaches using a heterogeneous dataset for qualitative analysis using DL, SVM, and DT methods in order to predict post-contrast information accurately without requiring contrast administration. While our initial results are modest, our investigation shows that this area still poses several open challenges and opportunities for further research.

### Limitations and future work

The main limitation of our study is that it included a relatively small number of patients from a single center. However, the study confirms the efficacy of ML methods and can improve our understanding of the diagnostic potentials of these emerging methods as well as data phenotypes that are yet to be standardized in CVD. Future work will focus on using novel DL architectures that combine both cine SAX image frames and the derived heterogenous variables to predict the extent and location of scar in the myocardium. To have a larger dataset for model training will involve automating the ground truth data preparation pipeline. Also, there is the need to consider risks associated with the applications when contrast is not administered when it should have been acquired and vice versa (i.e., the consequences of the false negatives and false positives).

## Conclusion

Cardiac magnetic resonance imaging has potential to benefit from practical and inexpensive methods in the emerging field of ML for diagnosing MI without the use of a contrast agent. We have presented some promising approaches using a heterogeneous dataset for qualitative analysis using ML methods in an attempt to predict contrast information accurately without requiring contrast administration. Our study provided an original contribution and development in this area, presenting new parameters, such as, rate of myocardial area change, optical flow and radiomics parameters, that could be considered biomarkers of the mechanics of myocardial disease. However, further studies that would improve the proposed methods and identify other parameters are needed, just as it would be necessary to develop such models on a larger population in order to validate the results and make it possible to reach acceptable prediction level that could make it possible and safe to avoid contrast administration in clinical CMR scans.

## Data availability statement

The datasets presented in this article are not readily available because restrictions apply to the availability of these raw data, which were used under license for the current study from Barts BioResource Institutional Review Board. Generated anonymised dataset can be made available from the authors upon reasonable request and with permission of Institutional Review Board of Barts BioResource, Barts Health NHS Trust, London, United Kingdom. Requests to access the datasets should be directed to SP, s.e.petersen@qmul.ac.uk.

## Ethics statement

Data used for this research were Barts BioResource – a local biorepository of Barts Heart Centre (Barts Health NHS Trust, London, United Kingdom) that holds data from prospectively consented (written) patients for cardiovascular research (Ethics REC reference: 14/EE/0007). All images were de-identified prior to analysis. The patients/participants provided their written informed consent to participate in this study.

## Author contributions

SP, MA, AL, KL, AY, MB, and PB conceived to the idea. MA, AA-K, ER, AS, AK, and SP developed the contouring method. MA led on the machine learning methodology and the main mathematical and statistical analysis, and drafted the first version of the manuscript. MA, AS, AK, KL, AY, MK, and SP contributed to the analysis. MA and AL advised on data governance and computing infrastructure. SP provided overall supervision. All authors contributed to the content, the writing of the final version or provided critical feedback.

## Abbreviations

CMR: cardiac magnetic resonance
CVD: cardiovascular disease
DT: decision tree
DL: deep learning
DSC: dice similarity coefficient (Dice score)
LGE: late gadolinium enhancement
LV: left ventricle
ML: machine learning
MI: myocardial infarction
PFI: permutation feature importance
ROI: region of interest
SAX: short axis
SI: signal intensity
SVM: support vector machines
TT: trigger time.
