# Supplementary material for: Predicting post-contrast information from contrast agent free cardiac MRI using machine learning: Challenges and methods
Source: Front Cardiovasc Med. 2022 Jul 27;9:894503. doi: 10.3389/fcvm.2022.894503 (PMC9426684; doi:10.3389/fcvm.2022.894503)
Supplement: Supplementary file 1 [file Data_Sheet_1.PDF]

Predicting Post-contrast Information from Contrast Agent Free Cardiac MRI using Machine Learning: Challenges and Methods

Supplementary Material

1. Lucas-Kanade Optical Flow Method

The goal of optical flow is to compute an approximation of 2-dimensional motion field from spatiotemporal data of image intensities (1). The basic assumption of optical flow algorithms is that the intensity constraint that can be written as follows:

$$I(x, y, t) = I(x + dx, y + dy, t + dt) \tag{S1}$$

where $I(x, y, t)$ is the intensity of the pixel at point (x, y) at time t, and $I(x + dx, y + dy, t + dt)$ is the intensity of that pixel at time $t + dt$. The Taylor series expansion formula can be applied to Equation (S1) assuming the pixel movement is small enough and ignoring the higher order terms to give the following:

$$\frac{\partial I}{\partial x}dx + \frac{\partial I}{\partial y}dy + \frac{\partial I}{\partial t}dt = 0 \tag{S2}$$

Equation (S2) can also be derived from Equation (S1) by assuming that the intensity is conserved, that is, $\frac{dI}{dt} = 0$ (2). Substituting Equation (S2) in Equation (S1) gives the following:

$$I_x v_x + I_y v_y + I_t = 0 \tag{S3}$$

where I_x, I_y and I_t represent partial derivatives of I with respect to x, y and t, respectively; v_x and v_y represent $\frac{dx}{dt}$ and $\frac{dy}{dt}$, respectively, and correspond to the horizontal and vertical optical flow velocities. Equation (S3) has two unknowns (v_x and v_y) constrained by one linear equation. Further constraints are therefore needed to solve for unknowns, and the weighted least square (WLS) method offers such constraints. The WLS can be used to compute the optical flow velocities by minimizing the squared error given as follows:

$$E(v_x, v_y) = \sum_{(x,y)\in\Omega} w(x, y)\left[I_x v_x + I_y v_y + I_t\right]^2 \tag{S4}$$

where Ω is the region (spatial neighbourhood) within which the constraints are applied and whose dimension is $\sqrt{N} \times \sqrt{N}$ (N is the number of points in Ω); $w(x, y)$ is the weighting function ("window" function) of Ω region. It is common to let $w(x, y)$ be Gaussian (2). The minimum of $E(v_x, v_y)$ can be obtained from its critical point by setting its derivatives with respect to v_x and v_y to zero; that is,

$$\frac{\partial E(v_x, v_y)}{\partial v_x} = \sum_{(x,y)\in\Omega} w(x, y)\left[v_x {I_x}^2 + v_y I_x I_y + I_x I_t\right] = 0$$

$$\frac{\partial E(v_x, v_y)}{\partial v_y} = \sum_{(x,y)\in\Omega} w(x, y)\left[v_y {I_y}^2 + v_x I_x I_y + I_y I_t\right] = 0$$

The last two equations can be written in matrix form as:

$$M\boldsymbol{v} = \boldsymbol{b} \tag{S5}$$

where

$M = \begin{bmatrix} \sum w\, {I_x}^2 & \sum w\, I_x I_y \\ \sum w\, I_x I_y & \sum w\, {I_y}^2 \end{bmatrix}$, $\boldsymbol{b} = -\begin{pmatrix} \sum w\, I_x I_t \\ \sum w\, I_y I_t \end{pmatrix}$, and $\boldsymbol{v} = \begin{pmatrix} v_x \\ v_y \end{pmatrix}$; and when M has a rank of 2, then WLS estimate $\widehat{\boldsymbol{v}}$ is as follows:

$$\widehat{\boldsymbol{v}} = M^{-1}\boldsymbol{b} \tag{S6}$$

The approach of estimating optical flow velocities using Equations (S4) – (S6) is often referred to as the Lucas-Kanade method.

2. Support vector machines method using radial basis kernel

Support vector machines (SVM) are a set of supervised ML methods that are effective for classification problems in high dimensional spaces. Given that the training set (x_i, y_i) of N number of samples with the labels $\boldsymbol{y}_i \in \{1, -1\}$ are associated with the feature vectors $\boldsymbol{x}_i \in \mathbb{R}^n$, $i = 1, 2, \ldots, L$ (alternatively, $\boldsymbol{y} \in \mathbb{R}^L$ such that $y_i \in \{1, -1\}$), the discriminant hyperplane is as follows (3,4):

$$f(\boldsymbol{x}) = \sum_{i=1}^{L} \boldsymbol{y}_i \boldsymbol{\alpha}_i \boldsymbol{K}(\boldsymbol{x}, \boldsymbol{x}_i) + b \tag{S7}$$

where $K(x, x_i)$ is a kernel function that maps the data set into different data space; the sign of $f(x)$ determines the membership of x; and α_i and b represent a support vector and bias, respectively. The objective of SVM is to find the optimal hyperplane (finding all the nonzero α_i and b) by solving the following optimization problem:

$$\min_{w, b, \xi} \quad w^T w + \lambda \sum_{i=1}^{L} \xi_i$$

$$\text{subject to} \quad y_i(w^T \phi(x_i) + b) \geq 1 - \xi_i\,, \quad \xi_i \geq 0$$

where ξ is the slack variable (allows some samples to be at a distance ξ_i from their correct margin boundary), λ (≥ 0) is the regularization (penalty) parameter, $w \in \mathbb{R}^n$, $b \in \mathbb{R}$, and $\phi(x_i)$ is the kernel function (a function that maps x_i into higher-dimensional space). The parameter λ is computed using the approach of specifying the class weights given by Equation (5) of the main manuscript wherein weights belonging to Class 0 and 1 are multiplied by 0.753 and 1.488, respectively. The kernel function $K(x, x_i) = \langle \phi(x), \phi(x_i) \rangle$ is chosen as the radial basis function that can be defined for two samples $\boldsymbol{x}$ and $\boldsymbol{x}'$ as follows:

$$K(\boldsymbol{x}, \boldsymbol{x}') = \exp\left(-\gamma \|x - x'\|^2\right)$$

where $\gamma = \frac{1}{L\, {\sigma_i}^2}$ (i.e. ${\sigma_i}^2$ is the variance of x_i and $\|x - x'\|^2$ is the Euclidean distance).

3. Decision tree method

Decision Trees (DT) are a type of supervised learning methods with the goal to create a model by learning simple decision rules inferred from the data features in a piecewise constant approximation manner (recursive partitioning), making the model simple to interpret. In a DT, the feature space is

recursively partitioned such that the samples with the same labels are grouped together. Given a node m whose data and number of samples are Q_m and N_m, respectively, and given the feature vectors $\boldsymbol{x}_i \in \mathbb{R}^n$, $i = 1, 2, \ldots, L$ and the label vector $\boldsymbol{y} \in \mathbb{R}^L$, then for the candidate split $\theta = (j, t_m)$ at m that has j number of features and a threshold of t_m, the data Q_m is partitioned into $Q_m^{left}(\theta)$ and $Q_m^{right}(\theta)$ subsets as follows (5):

$$Q_m^{left}(\theta) \; = \{(x, y) | x_j \leq t_m\}$$

$$Q_m^{right}(\theta) = Q_m \setminus Q_m^{left}(\theta)$$

('\' represents the set minus) and the optimal parameter (impurity) θ^* is given by:

$$\theta^* = argmin_\theta G(Q_m, \theta)$$

where $G(Q_m, \theta)$ is defined as follows:

$$G(Q_m, \theta) = \frac{N_m^{left}}{N_m} H\left(Q_m^{left}(\theta)\right) + \frac{N_m^{right}}{N_m} H(Q_m^{right}(\theta)) \tag{S8}$$

and $H()$ is the impurity (loss) function. The splitting is continued recursively for $Q_m^{left}(\theta^*)$ and $Q_m^{right}(\theta^*)$ until $N_m = 1$, or $N_m < N_{\min split}$, or the maximum allowable depth is reached. $N_{\min split}$ is the minimum number of samples required for a split at node m. A common choice of $H()$ is entropy, which is given as follows:

$$H() = -\sum_k p_{mk} \log(p_{mk}) \tag{S9}$$

(that is, $H()$ is $H(Q_m^{left}(\theta))$ and $H(Q_m^{right}(\theta))$ for left and right, respectively) and p_{mk} is defined as follows:

$$p_{mk} = \frac{1}{N_m} \sum_{y \in Q_m} 1(y = k)$$

where the k is the number of possible classification outcomes (of values 0, 1, 2, … $k - 1$) at node m (i.e. for binary classification, $k = 0, 1$) and $1(x)$ is the indicator function. In this study, to address our data imbalance, we have specified the class weights using the approach given by Equation (5) of the main manuscript wherein weights belonging to Class 0 and 1 are multiplied by 0.753 and 1.488, respectively. Also, we have chosen $N_{\min split} = 2$ and set the maximum allowable depth (useful in order to avoid overfitting) as 5.

Supplementary References

1. Barron JL, Fleet DJ, Beauchemin SS. Performance of optical flow techniques. *Int J Comput Vis* (1994) **12**:43–77.

2. Fleet D, Weiss Y. "Optical flow estimation," in *Handbook of mathematical models in computer vision* (Springer), 237–257.

3. Smola AJ, Schölkopf B. A tutorial on support vector regression. *Stat Comput* (2004) **14**:199–222.

4. Fan R-E, Chang K-W, Hsieh C-J, Wang X-R, Lin C-J. LIBLINEAR: A library for large linear classification. *J Mach Learn Res* (2008) **9**:1871–1874.

5. Hastie T, Tibshirani R, Friedman JH, Friedman JH. *The elements of statistical learning: data mining, inference, and prediction*. Springer (2009).
